# Metal Dimers‐Doped h‐BN Structures as Novel Toxic Gases Sensors With Enhanced Sensitivity Properties: An ADFT Study

**DOI:** 10.1002/jcc.70062

**Published:** 2025-02-11

**Authors:** H. Cruz‐Martínez, H. Rojas‐Chávez, L. Santiago‐Silva, L. López‐Sosa, P. Calaminici

**Affiliations:** ^1^ Tecnológico Nacional de México Instituto Tecnológico del Valle de Etla Oaxaca Mexico; ^2^ Tecnológico Nacional de México Instituto Tecnológico de Tláhuac II Mexico City Mexico; ^3^ Departamento de Química Cinvestav Mexico City Mexico

**Keywords:** auxiliary density functional theory (ADFT), defective h‐BN, metal dimers, sensitivity, stability

## Abstract

Toxic gases monitoring and detection are fundamental to lessening public health problems. Therefore, in this work, to explore emergent sensor materials, 3*d*‐metal dimers‐doped hexagonal boron nitride (h‐BN) structures were investigated employing auxiliary density functional theory (ADFT) as novel CO and NO gas sensors. Firstly, the stabilities of Co_2_, Ni_2_, and Cu_2_ dimers deposited on defective h‐BN were determined. Then, sensitivities of 3*d*‐metal dimers‐doped h‐BN structures towards the NO and CO gases were investigated. It was found that the interaction energies of these 3*d*‐metal dimers embedded on defective h‐BN are higher than those deposited on pristine h‐BN structure, which indicates that the 3*d*‐metal dimers exhibit good stability on defective h‐BN. Moreover, this work demonstrated that the CO and NO adsorption energies on 3*d*‐metal dimers‐doped h‐BN structures are higher than those computed in the literature for pristine h‐BN structure. Consequently, the here considered 3*d*‐metal dimers‐doped h‐BN structures can be good candidates for toxic CO and NO gas detection.

## Graphical abstract

The development of materials capable of detecting high toxicity gases is key for avoiding human health risks. In this work, 3*d*‐metal dimers‐doped hexagonal boron nitride (h‐BN) structures were investigated employing density functional theory as novel CO and NO sensors. The CO and NO adsorption energies on these structures were higher than those computed in the literature for pristine h‐BN structure. Consequently, the 3*d*‐metal dimers‐doped h‐BN structures can be good candidates for detection of these gases.

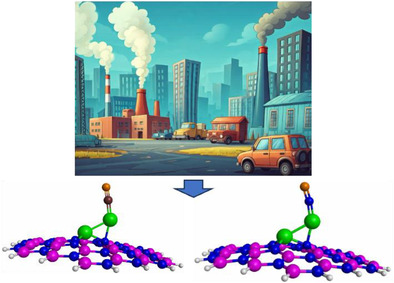

## 1 Introduction

The development of materials capable of detecting pollutants such as high toxicity gases is key for avoiding human health risks [1, 2, 3, 4]. Health problems due to air contamination can cause several kinds of damage. For instance, eye irritation, and even death caused by poisoning. One of the harmful gases is carbon monoxide (CO) which is under atmospheric conditions, a colorless and odorless gas. Depending on its concentration, it has adverse effects on humans. It has been reported that the exposure to CO levels above 150–200 ppm can cause unconsciousness, disorientation, and death [5]. Even more, it has been documented that, during the hurricane season, people have an increased generator usage due to the power outages, which causes unintentional CO poisoning deaths [6].

Under this context, the literature also highlights that nitrogen oxides (NO_x_) emissions cause environmental air pollution which give way to a variety of health issues, particularly in the field of respiration for human health, e.g., respiratory infections, respiratory tract, among others. Moreover, NO_x_ gases are strongly correlated with the incidence of different types of cancer [7]. NO_x_ are also considered as one of the primary greenhouse gases responsible for reducing air quality [8, 9, 10].

Recently, two‐dimensional (2D) structures have been investigated to detect and monitor toxic gases due to their high specific surface area [11, 12, 13]. After the utilization of graphene‐based materials for toxic gases detection, investigations have focused on different graphene‐like structures. For instance, the hexagonal boron nitride (h‐BN) structure is a promising toxic gas sensor [14, 15, 16]. However, it has been reported that the 2D structures have limited reactivity for toxic gases [17, 18, 19]. Therefore, various approaches have been explored to modify the pristine h‐BN reactivity, such as doping [20, 21]. In this direction, there is a sizeable amount of evidence where doped h‐BN has shown promising results for the detection of different molecules, highlighting the use of metal‐doped h‐BN structures [22, 23, 24, 25, 26, 27, 28, 29, 30, 31, 32, 33]. For instance, the NO and NO_2_ adsorptions were investigated on Rh‐doped h‐BN structure employing density functional theory (DFT) calculations [28], exhibiting for this structure good results for the detection of these gases. In another study, the CO and NO adsorptions on Si‐doped h‐BN were investigated using DFT [29]. The Si‐doped h‐BN structure exhibited a higher reactivity than pristine *h*‐BN for CO and NO detection. Also, the CO and NO adsorption properties on Ti‐, V‐, Cr‐, Mn‐, Fe‐ Co‐, Ni‐, Cu‐, Pd‐, Ag‐, Pt‐, Au‐doped h‐BN were investigated using first‐principle calculations [30]. The CO adsorption energies calculated on metal‐doped h‐BN exhibited a higher reactivity than the pristine *h*‐BN. Additionally, the NO adsorption on Co‐, Ni‐, Cu‐, Pd‐, Pt‐, Au‐doped h‐BN was more favored in comparison to pristine *h*‐BN. Furthermore, the Fe‐ Co‐, Ni‐, Ru‐, Rh‐, Pd‐, Os‐, Ir‐, Pt‐doped h‐BN structures were investigated as CO sensors employing DFT calculations, exhibiting promising results [31]. In another study, the CO, NO, NO_2_, and N_2_O adsorptions on Pd‐ and Ni‐doped h‐BN structures were studied, showing good sensitivities towards these gases [32]. Recently, the CO adsorption on Co‐doped h‐BN structure was investigated using DFT calculations [33]. The Co‐doped h‐BN structure exhibited a higher reactivity than pristine *h*‐BN for CO detection. It was observed that the metal atom‐doped h‐BN structures are better candidates for CO and NO_x_ detection than the pristine h‐BN structure [28, 29, 30, 31, 32, 33]. Even though the current results of the doped h‐BN structures provide some insights on the sensitivity towards toxic gases, more doped h‐BN systems should be studied for the detection of such gases. Therefore, here we study 3*d*‐metal dimers‐doped h‐BN structures as novel CO and NO sensors using auxiliary DFT (ADFT) calculations. First, the stabilities of Co_2_, Ni_2_, and Cu_2_ on defective h‐BN were investigated. After, the sensitivities of Co_2_‐, Ni_2_‐, and Cu_2_‐doped h‐BN structures for CO and NO detection were computed.

The manuscript is organized as follows. The computational details and the information about the employed model structures for this investigation are given in the next section. The results obtained are presented and discussed in section 3. Finally, the conclusions are resumed in the last section.

## 2 Methodology

### 2.1 Electronic Structure Method and Calculation Details

All electronic structure computations were performed using the ADFT method as implemented in the deMon2k code [34]. The variational fitting method was used to compute the Coulomb energy contribution [35]. The exchange and correlation rev‐PBE functional was employed for all calculations [36]. The Cu atoms were described with a TZVP‐GGA basis set, and for all the others remaining atoms an DZVP‐GGA basis set was employed [37]. All calculations were carried out using the GEN‐A2* auxiliary‐function‐set [37]. The restricted‐open‐shell Kohn‐Sham approximation was used for open‐shell systems to avoid spin contaminations [38]. All structures were optimized in the delocalized internal coordinates using the quasi‐Newton method [39]. After that, the optimized most stable structures were characterized by frequency calculations. It is important to highlight that the computational methodology used in this investigation has been validated in our previous studies and the obtained computational results exhibited good agreement with the experimental data [40, 41, 42].

### 2.2 Models

**FIGURE 1:**
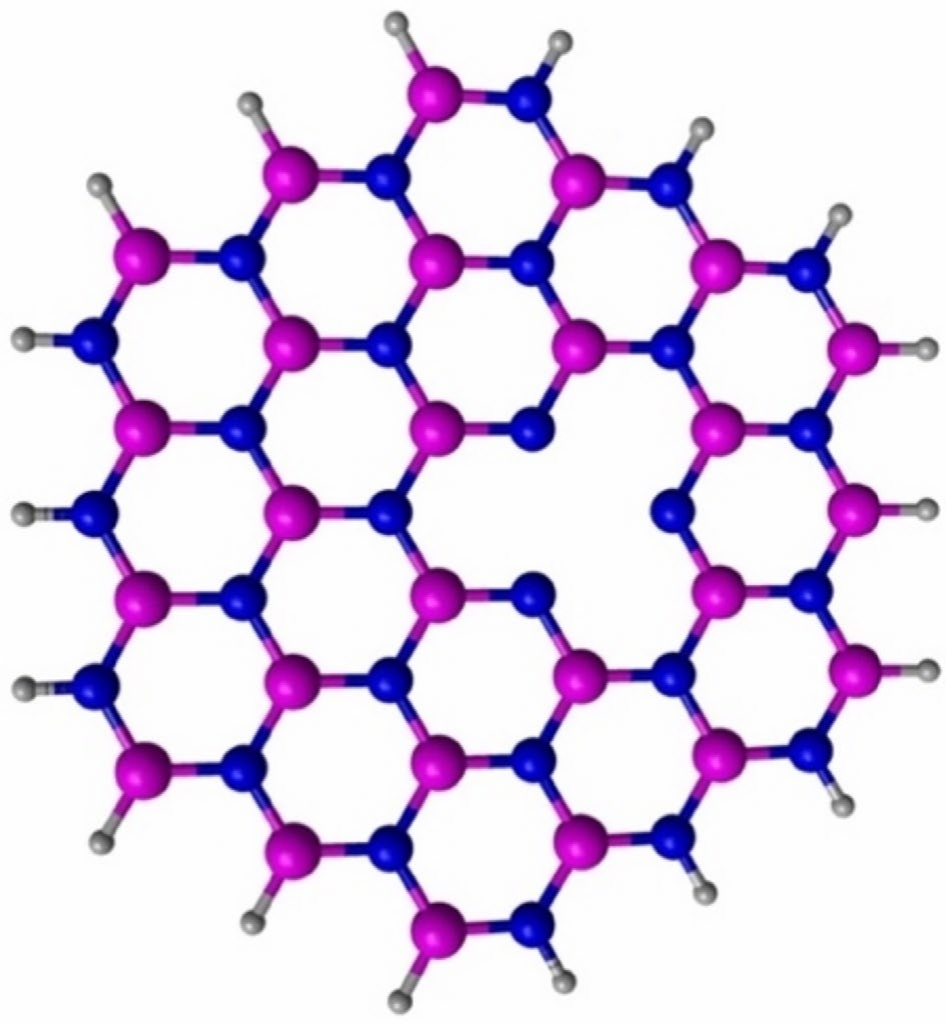
Illustration of the defective h‐BN structure. The white, blue, and pink spheres represent H, N, and B atoms, respectively.

To study the CO and NO adsorptions on the 3*d*‐metal dimers‐doped h‐BN structures, the B_27_N_27_H_18_ structure was used as a model for pristine h‐BN. To dope the pristine h‐BN structure with the metal dimers, a B atom was removed from the center of the h‐BN structure as shown in Figure 1. We have focused our work on this specific type of vacancy in h‐BN structures, because this type of vacancy has been obtained by experimental groups [43, 44]. Then, the metal dimers are deposited on defective h‐BN. We have doped the defective h‐BN structure with metal dimers, since it has been demonstrated that metal dimers‐modified structures present promising properties for various applications [45, 46]. To obtain the most stable interaction between the metal‐dimers and the h‐BN, four initial interactions Figure (SI‐1) were considered and subsequently optimized with different spin multiplicities. We have selected these four initial structures, since these were the four different structures that we found for the metal dimers near the vacancy of defective h‐BN structures, since as is well known in the literature, the vacancy is the active site of these types of structures. The interaction energies (*E*_int_) between the metal dimers and defective h‐BN were obtained using a previously reported equation [47] as:

$$E_{\mathrm{int}}=E_{\text{Metal}-\text{dimer}-\text{doped}\;h-\mathrm{BN}}-\left(E_{\text{Metal}-\text{dimer}}+E_{\text{defective}\;h-\mathrm{BN}}\right)$$

where $E_{\text{Metal}-\text{dimer}-\text{doped}\;h-\mathrm{BN}}$ is the total energy of the metal‐dimer‐doped h‐BN structures, $E_{\text{Metal}-\text{dimer}}$ y $E_{\text{defective}\;h-\mathrm{BN}}$ are the total energy calculated as a single point of the free‐standing metal‐dimer and defective h‐BN after being geometrically optimized, respectively.

To investigate the most stable adsorption of the CO and NO molecules on 3*d‐*metal dimers‐doped h‐BN structures, different CO and NO adsorption modes (top and bridge) on these structures were proposed and optimized. The adsorption energies ($E_{\mathrm{ads}})$ of the gases on the metal‐dimers‐doped h‐BN materials were calculated using the following equation:

$$E_{\mathrm{ads}}=E_{\mathrm{Gas}/\text{Metal}-\text{dimer}-\text{doped}\;h-\mathrm{BN}}-\left(E_{\mathrm{Gas}}+E_{\text{Metal}-\text{dimer}-\text{doped}\;h-\mathrm{BN}}\right)$$

where $E_{\mathrm{Gas}/\text{Metal}-\text{dimer}-\text{doped}\;h-\mathrm{BN}}$ is the total energy of the gas molecules adsorbed on metal‐dimer doped h‐BN materials, $E_{\text{Metal}-\text{dimer}-\text{doped}\;h-\mathrm{BN}}$ is the total energy of metal‐dimer doped h‐BN, and $E_{\mathrm{Gas}}$ is the total energy of isolated gas molecules.

## 3 Results and Discussion

### 3.1 Stability of Metal Dimers Deposited on Defective h‐BN

**FIGURE 2:**
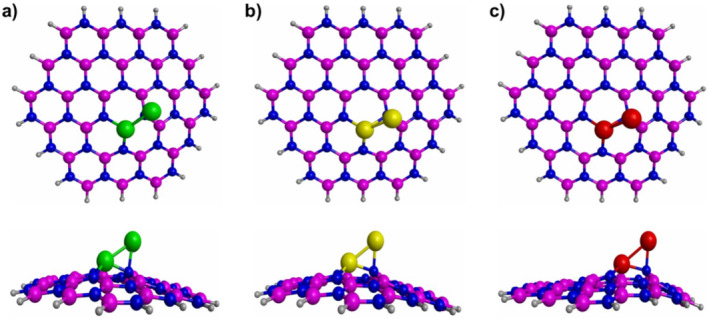
The most stable interactions between the metal dimers and the defective h‐BN. (a) Co_2_‐doped h‐BN, (b) Ni_2_‐doped h‐BN, (c) Cu_2_‐doped h‐BN. The white, blue, pink, green, yellow and red spheres represent H, N, B, Co, Ni, and Cu atoms, respectively.

Figure 2 reports the most stable interactions between the metal dimers and the defective h‐BN. For the three studied composites, it is observed that one of the metal dimers is deposited on the vacancy of the h‐BN structure. While the other metal atom is anchored to one N atom of the h‐BN structure. It is important to notice that when the metal dimers are deposited on the defective h‐BN structure, a structural modification is observed in the defective h‐BN structure. This effect has already been observed in literature, and it has been attributed to the presence of the doping atoms [27, 29]. To confirm that the structures reported in Figure 2 are stable, frequency analyses were conducted. All computed first frequencies are real (Table 1), assuring the minima nature of these structures.

**TABLE 1:** First frequencies, interaction energies (*E*_int_), charge transfer and HOMO–LUMO gap of the metal dimers deposited on defective h‐BN structure.

Systems	First frequencies (cm^−1^)	*E*_int_ (eV)	Charge (*e*)	HOMO–LUMO gap (eV)
Co_2_/defective‐h‐BN	6	−8.92	1.23	1.03
Ni_2_/defective‐h‐BN	25	**−**8.81	1.12	0.39
Cu_2_/defective‐h‐BN	13	−6.43	1.21	0.52

**FIGURE 3:**
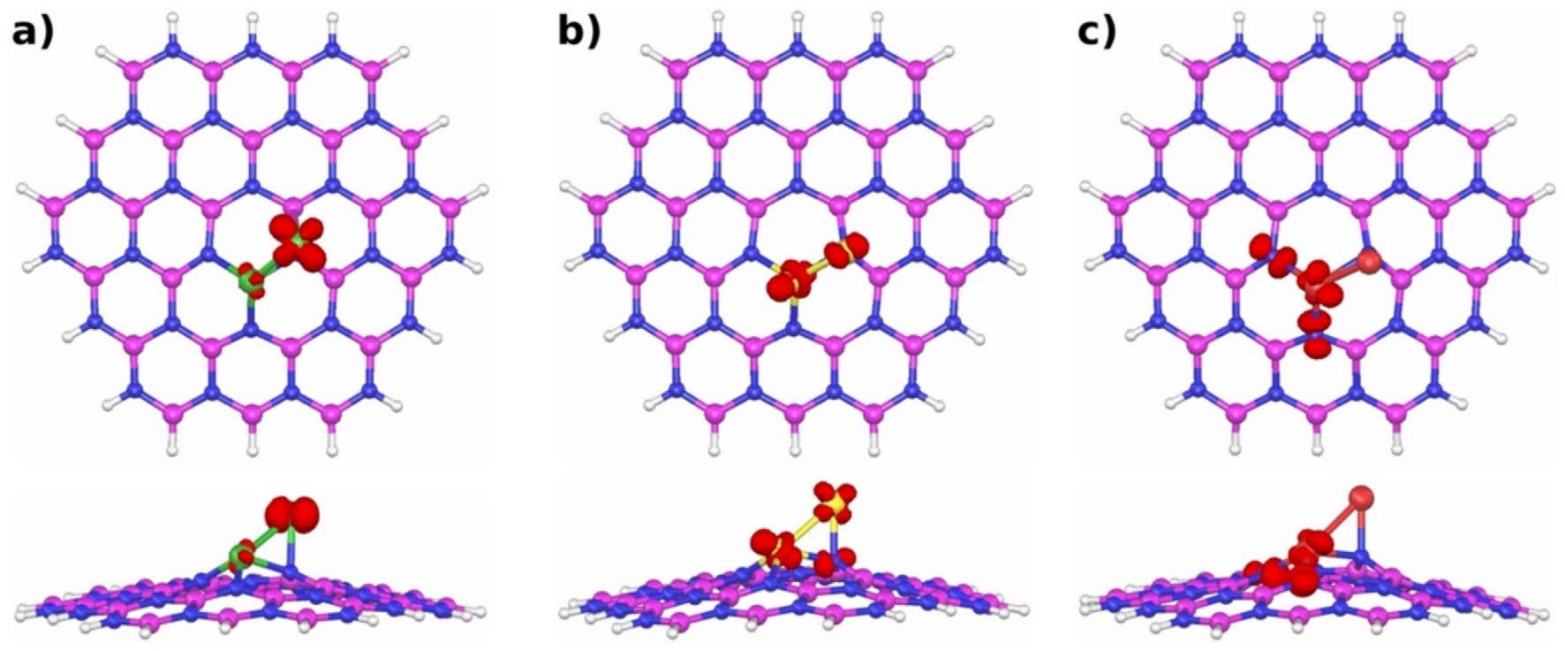
Spin density plots of the 3*d*‐metal dimers‐doped h‐BN structures. (a) Co_2_‐doped h‐BN, (b) Ni_2_‐doped h‐BN, (c) Cu_2_‐doped h‐BN. The white, blue, pink, green, yellow and red spheres represent H, N, B, Co, Ni and Cu atoms, respectively.

Concerning the spin multiplicity of the metal dimers‐doped h‐BN structures, it was observed that all three systems exhibit a doublet spin multiplicity, which indicates that these are open‐shell systems. Therefore, it is essential to understand where their spin density is mostly allocated. The results of the computed spin density distributions are graphically illustrated in Figure 3. As can be seen from Figure 3, for the Co_2_‐doped h‐BN structure, the spin density is mostly located on a Co atom (Figure 3a). Whereas for the Ni_2_‐doped h‐BN structure, the spin density is located on the two Ni atoms and one N atom (Figure 3b). Finally, for the Cu_2_‐doped h‐BN structure, the spin density is located on one Cu atom and on two N atoms (Figure 3c).

To better understand the interaction between the 3*d*‐metal dimers and the defective h‐BN structure, the *E*_int_ and Bader charge transfer were computed. The results obtained are summarized in Table 1. It is observed that as the atomic number of the dimer atoms decreases, the *E*_int_ tends to increase (see Table 1). Also, the calculated *E*_int_ is greater than that reported in the literature for metal atoms [48] o clusters [49] embedded on pristine h‐BN. Therefore, it is deduced that the defective h‐BN structure is a good material to stabilize the 3*d*‐metal dimers. Analyzing the Bader charge transfer, we notice that the considered 3*d*‐metal dimers transfer charge to the defective h‐BN structure, since they exhibit a positive charge (see Table 1). Moreover, the metal dimer atoms transfer more than 1 *e* of charge to the h‐BN structure as shown in Table 1. Finally, to obtain the reactivity of the metal dimers‐doped h‐BN structures, the energy differences between the frontier orbitals (HOMO–LUMO gap) were computed and the obtained results are presented in the last column of Table 1. We notice that the investigated systems exhibit a HOMO–LUMO gap close to or less than 1 eV, evidencing a promising reactivity. Also, the 3*d*‐metal dimers‐doped h‐BN structures exhibit better reactivity that the B_27_N_27_H_18_ structure, since a HOMO–LUMO gap larger than 5 eV has been reported in the literature for this structure [50, 51].

### 3.2 CO Adsorption on 3*d*‐Metal Dimers‐Doped h‐BN Structures

**FIGURE 4:**
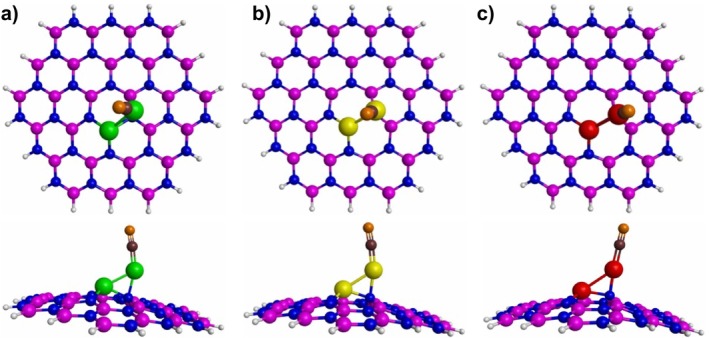
Most stable adsorption sites of the CO molecule on the 3*d*‐metal dimers‐doped h‐BN structures. (a) CO adsorption on Co_2_‐doped h‐BN, (b) CO adsorption on Ni_2_‐doped h‐BN, (c) CO adsorption on Cu_2_‐doped h‐BN. The white, blue, pink, green, yellow, red, brown, and orange spheres represent H, N, B, Co, Ni, Cu, C, and O atoms, respectively.

The most stable CO adsorptions on 3*d‐*metal dimers‐doped h‐BN structures are illustrated in Figure 4. It is observed that the CO adsorption occurs via the C atom on a metal atom of the 3*d‐*metal dimers‐doped h‐BN structures, which is consistent with results previously reported [29, 31, 33]. Concerning the bond lengths, all bond metal‐C lengths are shorter than 2 Å, which suggests that there is a strong interaction between the CO molecule and the 3*d‐*metal dimers‐doped h‐BN structures. The CO molecule exhibited a bond length elongation when this is adsorbed on the 3*d‐*metal dimers‐doped h‐BN structures, which can be associated with the transfer of charge from the 3*d‐*metal dimers‐doped h‐BN structures to the CO molecule (see Table 2). It is observed that as the atomic number of the dimer atoms decreases, the charge transfer from 3*d‐*metal dimers‐doped h‐BN structures to CO molecule tends to increase (see Table 2). According to several authors the binding of CO molecule to 3*d* metals typically occurs via a charge donation from the CO bonding sigma orbital to the metal's orbital, which in turn donates back charge to the unoccupied pi orbitals of CO [52, 53]. From the computed negative charges on CO it follows that the pi‐back donation is preponderant for the three studied metal dimers.

**TABLE 2:** The metal–C and C–O bond lengths, adsorption energies (*E*_ads_) and charge transfer between the CO molecule and 3*d*‐metal‐dimers‐doped h‐BN structures.

Systems	Bond lengths (Å)		*E*_ads_ (eV)	CO charge (*e*)
	M–C	C–O		
CO	—	1.156	—	—
Co_2_/defective‐h‐BN	1.738	1.181	−2.62	−0.35
Ni_2_/defective‐h‐BN	1.929	1.176	−2.64	−0.29
Cu_2_/defective‐h‐BN	1.790	1.163	−2.05	−0.10

The *E*_ads_ were also calculated since they have been widely used to study the sensitivity of various materials towards toxic gases [29, 31, 33]. It is observed that the *E*_ads_ are higher than those calculated for the CO molecule adsorbed on pristine h‐BN structure, since in the literature for the adsorption of CO on h‐BN structure values less than −0.3 eV have been calculated [29, 31, 33, 54]. Also, computed *E*_ads_ of CO on metal dimers‐doped h‐BN structures were higher than those calculated on Co‐, Ni‐, Cu‐doped h‐BN structures [30, 31]. The *E*_ads_ of CO on the Co_2_‐ and Ni_2_‐doped h‐BN structures are very similar, being of −2.62 and −2.64 eV respectively. These two systems exhibited a higher *E*_ads_ of CO than the Cu_2_‐doped h‐BN structure (see Table 2). Based on the calculated values of the *E*_ads_, it can be inferred that the 3*d‐*metal dimers‐doped h‐BN structures here studied may exhibit good sensitivity towards the CO molecule, since higher *E*_ads_ can be associated with a higher sensitivity [29, 31, 33].

### 3.3 NO Adsorption on 3*d*‐Metal Dimers‐Doped h‐BN Structures

**FIGURE 5:**
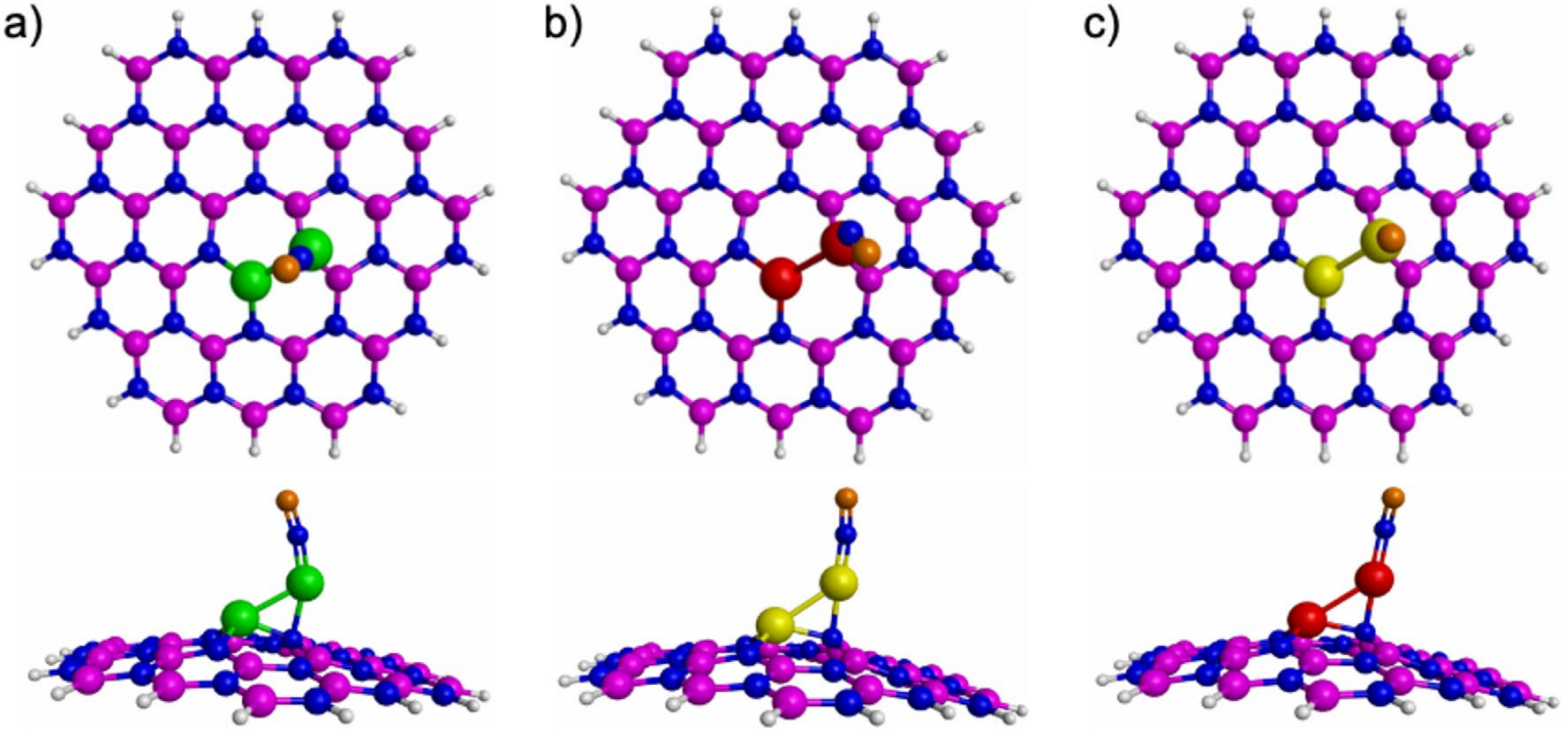
Most stable adsorption sites of the NO molecule on the 3*d*‐metal dimers‐doped h‐BN structures. (a) NO adsorption on Co_2_‐doped h‐BN, (b) NO adsorption on Ni_2_‐doped h‐BN, (c) NO adsorption on Cu_2_‐doped h‐BN. The white, blue, pink, green, yellow, red, and orange spheres represent H, N, B, Co, Ni, Cu, and O atoms, respectively.

The most stable NO adsorptions on 3*d‐*metal dimers‐doped h‐BN structures are illustrated in Figure 5. It is observed that the NO adsorption is proceeding via a N atom on a metal atom of the 3*d‐*metal dimers‐doped h‐BN structures, which agrees with the reported literature [29, 32]. Analyzing the obtained bond lengths, we notice that the bond metal‐N lengths are shorter than 2 Å. This result can be associated with a strong interaction between the NO molecule and the 3*d‐*metal dimers‐doped h‐BN structures.

As in the case of the NO molecule, when the NO molecule is adsorbed on the 3*d‐*metal dimers‐doped h‐BN structures, an elongation of the N‐O bond length can be observed, which can be attributed to the charge transfer from the 3*d‐*metal dimers‐doped h‐BN structures to the NO molecule as it can be seen from Table 3. It is observed that as the atomic number of the dimer atoms decreases, the charge transfer from 3*d‐*metal dimers‐doped h‐BN structures to NO molecule tends to increase (see Table 3). It is observed that in this case the values of the *E*_ads_ are higher than those reported for the NO molecule adsorbed on pristine h‐BN structure. In fact, in the literature for the NO adsorption on h‐BN structure, *E*_ads_ values less than −0.3 eV have been calculated [29].

**TABLE 3:** The metal–C and N–O bond lengths, adsorption energies (*E*_ads_) and charge transfer between the NO molecule and 3*d*‐metal dimers‐doped h‐BN structures.

Systems	Bond lengths (Å)		*E*_ads_ (eV)	NO charge (*e*)
	M–C	N–O		
NO	—	1.179	—	—
Co_2_/defective‐h‐BN	1.612	1.191	−3.48	−0.41
Ni_2_/defective‐h‐BN	1639	1.188	−2.99	−0.36
Cu_2_/defective‐h‐BN	1.791	1.204	−1.95	−0.33

Also, we notice that the calculated values of the *E*_ads_ of NO on the 3*d‐*metal dimers‐doped h‐BN structures are higher than those reported for the NO molecule adsorbed h‐BN structure with B vacancy [55]. This finding suggests that metal dimers act as active sites for CO adsorption.

Finally, for the studied systems, the following trend for the here computed *E*_ads_ values is observed: Co_2_/defective‐h‐BN>Ni_2_/defective‐h‐BN>Cu_2_/defective‐h‐BN. Based on these *E*_ads_ values, it can be therefore inferred that the 3*d‐*metal dimers‐doped h‐BN structures may exhibit good sensitivity towards the NO molecule.

## 4 Conclusions

The Co_2_‐, Ni_2_‐, and Cu_2_‐doped h‐BN structures were studied as novel CO and NO gas sensors using the generalized gradient approximation and employing auxiliary density functional theory. It is observed that the *E*_int_ of the 3*d*‐metal dimers embedded on defective h‐BN are higher than those deposited on pristine h‐BN structure. This finding indicates that the 3*d*‐metal dimers exhibit good stabilities on defective h‐BN. The calculated *E*_int_ presented the following trend: Co_2_‐doped h‐BN>Ni_2_‐doped h‐BN>Cu_2_‐doped h‐BN. The investigated composites exhibit a HOMO–LUMO gap close or smaller than 1 eV, evidencing a promising reactivity of these systems. The *E*_ads_ of CO and NO molecules on the 3*d*‐metal dimers‐doped h‐BN structures are higher than those computed in the literature for the pristine h‐BN structure. Consequently, based on the obtained results the here studied 3*d*‐metal dimers‐doped h‐BN structures can be considered as potential good candidates for toxic CO and NO gas detections.

## Conflicts of Interest

The authors declare no conflicts of interest.

## Supporting information


**Data S1.** Supporting Information.

## Data Availability

The data that supports the findings of this study are available in the supporting Information of this article.
