# Supplementary material for: Metal Dimers‐Doped h‐BN Structures as Novel Toxic Gases Sensors With Enhanced Sensitivity Properties: An ADFT Study
Source: J Comput Chem. 2025 Feb 11;46(5):e70062. doi: 10.1002/jcc.70062 (PMC11827289; doi:10.1002/jcc.70062)
Supplement: Supplementary file 1 — Data S1. Supporting Information. [file JCC-46-0-s001.pdf]

Metal dimers-doped h-BN structures as novel toxic gases sensors with enhanced sensitivity properties: An ADFT study

H. Cruz-Martínez[1, *], H. Rojas-Chávez[2], L. Santiago-Silva[1],

L. López-Sosa[3], P. Calaminici[3, *]

[1]Tecnológico Nacional de México, Instituto Tecnológico del Valle de Etla, Abasolo S/N, Barrio del Agua Buena, Santiago Suchilquitongo, Oaxaca 68230, Mexico.

[2]Tecnológico Nacional de México, Instituto Tecnológico de Tláhuac II, Camino Real 625, Col. Jardines del Llano, San Juan Ixtayopan, Alcaldía Tláhuac, CDMX 13550, Mexico.

[3]Departamento de Química, Cinvestav, Av. Instituto Politécnico Nacional 2508, Gustavo A. Madero, 07360, Mexico City, Mexico

1.- Initial structures of the interaction between the metal dimers and the defective h-BN.

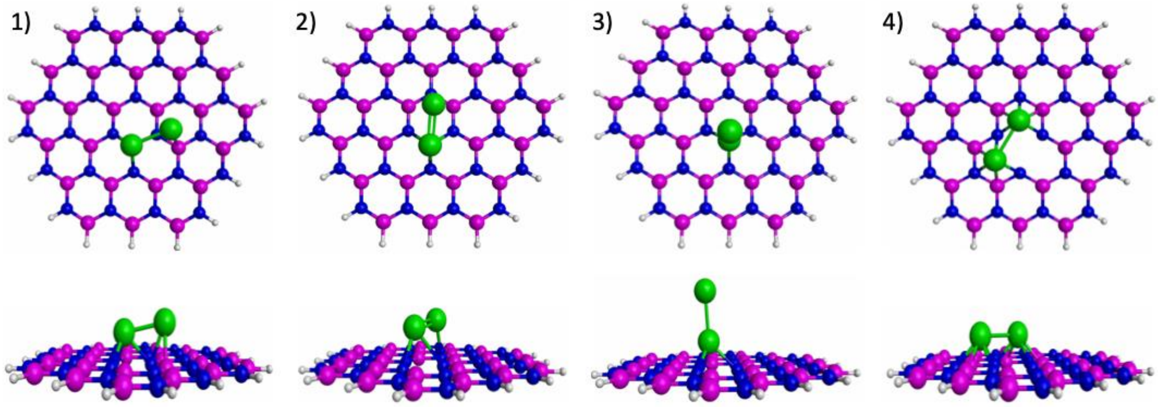

Figure SI-1. Four initial structures of the interaction between the metal dimers and the defective h-BN. The white, blue, pink, green spheres represent H, N, B, and metal atoms, respectively.

2.- Cartesian coordinates of the initial structures of the interaction between the metal dimers and the defective h-BN reported in the Figure SI-1.

Structure 1:

N	-0.123944	0.035434	1.307903
B	-0.125217	0.036750	-0.153331
N	1.135080	0.036443	-0.842379
B	2.415417	0.034884	-0.153531
N	3.684133	0.034584	-0.849834
B	4.952892	0.033049	-0.153570
N	6.233219	0.032705	-0.842400
B	7.493528	0.031129	-0.153368
N	7.492270	0.029817	1.307872
B	8.750572	0.028165	2.037853
N	8.753516	0.026868	3.492573
B	10.019603	0.025231	4.222106
N	9.986110	0.023985	5.658093
B	8.749379	0.024254	6.422464
N	8.718095	0.022950	7.869367
B	7.480777	0.023248	8.619997
N	7.437200	0.021962	10.073273
B	6.210385	0.022253	10.820260
N	4.945504	0.023898	10.088643
B	4.949775	0.025189	8.618808
N	3.684203	0.026755	7.888161
Co	3.603708	1.416658	6.452302
N	2.418357	0.029695	5.693888
B	1.152238	0.029911	6.424954
N	1.155328	0.028650	7.887213
B	-0.112358	0.028997	8.620036
N	-0.068784	0.027740	10.073299
B	1.158042	0.026151	10.820305
N	2.422912	0.025835	10.088661
B	2.418622	0.027115	8.618846
B	1.151101	0.033835	2.039127
N	1.151104	0.032510	3.500463
B	2.418087	0.030974	4.231998
N	3.684179	0.030689	3.501238
B	4.950284	0.029106	4.231979
N	6.217230	0.028791	3.500445
B	7.482772	0.027215	4.231172
N	7.480925	0.025925	5.691064
B	6.216152	0.026140	6.424941
N	6.213078	0.024822	7.887164
B	-1.382221	0.035696	2.037926
N	-1.385158	0.034455	3.492657

B	-2.651227	0.034753	4.222183
N	-2.617767	0.033456	5.658147
B	-1.381028	0.031822	6.422547
N	-1.349694	0.030534	7.869427
N	2.416284	0.033562	1.310729
B	3.684173	0.031954	2.039139
N	4.952056	0.031706	1.310675
B	6.217191	0.030133	2.039065
N	4.950012	0.027782	5.693862
B	3.684210	0.024204	10.813499
B	-0.114401	0.032850	4.231253
N	-0.112534	0.031496	5.691131
H	6.252052	0.033617	-1.861965
H	8.528660	0.030938	-0.770272
H	9.793468	0.027954	1.435698
H	11.071391	0.024913	3.634043
H	10.878470	0.022804	6.151591
H	-3.703025	0.036072	3.634112
H	8.310761	0.020830	10.599355
H	6.227054	0.021238	12.025185
H	-1.160403	0.038041	-0.770158
H	-2.425101	0.037009	1.435779
H	3.684225	0.023179	12.017769
H	1.141364	0.025155	12.025231
H	-0.942382	0.027914	10.599324
H	-2.232733	0.030786	8.379282
H	-3.510132	0.033682	6.151649
H	1.116241	0.037346	-1.861950
H	3.684181	0.035509	-1.869508
H	9.601175	0.021813	8.379138
Co	5.725346	1.867545	5.574996

Structure 2:

N	-0.123944	0.035434	1.307903
B	-1.382221	0.035696	2.037926
N	-1.385158	0.034455	3.492657
B	-2.651227	0.034753	4.222183
N	-2.617767	0.033456	5.658147
B	-1.381028	0.031822	6.422547
N	-1.349694	0.030534	7.869427
B	-0.112359	0.029000	8.620038
N	-0.068784	0.027743	10.073301
B	1.158042	0.026154	10.820306
N	2.422912	0.025838	10.088663

B	2.418620	0.027117	8.618847
N	1.155327	0.028652	7.887215
B	1.152237	0.029913	6.424956
N	-0.112534	0.031496	5.691131
B	-0.114401	0.032850	4.231253
N	1.151104	0.032510	3.500463
B	2.418087	0.030974	4.231998
N	2.418356	0.029697	5.693890
N	3.684201	0.026757	7.888162
B	4.949773	0.025191	8.618809
N	4.945504	0.023901	10.088644
B	3.684210	0.024207	10.813500
B	-0.125217	0.036750	-0.153331
N	1.135080	0.036443	-0.842379
B	2.415417	0.034884	-0.153531
N	3.684133	0.034584	-0.849834
B	4.952892	0.033049	-0.153570
N	6.233219	0.032705	-0.842400
B	7.493528	0.031129	-0.153368
N	7.492270	0.029818	1.307872
B	8.750572	0.028166	2.037853
N	8.753514	0.026870	3.492573
B	10.019601	0.025233	4.222106
N	9.986108	0.023987	5.658093
B	8.749378	0.024257	6.422464
N	7.480925	0.025926	5.691064
B	7.482772	0.027216	4.231172
N	6.217230	0.028792	3.500445
B	6.217191	0.030134	2.039065
N	4.952056	0.031706	1.310675
B	3.684173	0.031954	2.039139
N	3.684179	0.030689	3.501238
B	4.950284	0.029106	4.231979
N	4.950012	0.027782	5.693862
B	6.216152	0.026140	6.424941
N	6.213078	0.024822	7.887164
B	7.480777	0.023250	8.619997
N	8.718093	0.022953	7.869367
B	1.151101	0.033835	2.039127
N	2.416284	0.033562	1.310729
N	7.437200	0.021965	10.073273
B	6.210385	0.022256	10.820261
H	6.252052	0.033617	-1.861965
H	8.528660	0.030939	-0.770272
H	9.793468	0.027956	1.435698
H	11.071389	0.024916	3.634044
H	10.878468	0.022808	6.151592

H	-3.703025	0.036072	3.634112
H	8.310761	0.020834	10.599355
H	6.227054	0.021241	12.025186
H	-1.160403	0.038041	-0.770158
H	-2.425101	0.037009	1.435779
H	3.684225	0.023182	12.017770
H	1.141364	0.025158	12.025232
H	-0.942382	0.027915	10.599326
H	-2.232733	0.030786	8.379282
H	-3.510132	0.033682	6.151649
H	1.116241	0.037346	-1.861950
H	3.684181	0.035509	-1.869508
H	9.601173	0.021818	8.379138
Co	3.701442	1.902748	6.778101
Co	3.879418	2.165215	4.601077

Structure 3:

N	-0.123944	0.035434	1.307903
B	1.151101	0.033835	2.039127
N	2.416284	0.033562	1.310729
B	3.684173	0.031954	2.039139
N	3.684179	0.030689	3.501238
B	4.950284	0.029106	4.231979
N	4.950012	0.027782	5.693862
B	6.216152	0.026140	6.424941
N	6.213078	0.024822	7.887164
B	7.480777	0.023250	8.619997
N	7.437200	0.021965	10.073273
B	6.210385	0.022256	10.820261
N	4.945504	0.023901	10.088644
B	3.684210	0.024207	10.813500
N	2.422912	0.025838	10.088663
B	2.418621	0.027117	8.618847
N	3.684202	0.026757	7.888162
B	4.949774	0.025191	8.618809
B	-1.382221	0.035696	2.037926
N	-1.385158	0.034455	3.492657
B	-0.114401	0.032850	4.231253
N	1.151104	0.032510	3.500463
B	2.418087	0.030974	4.231998
N	2.418356	0.029697	5.693890
B	1.152237	0.029913	6.424956
N	-0.112534	0.031496	5.691131
B	-1.381028	0.031822	6.422547

N	-1.349694	0.030534	7.869427
B	-0.112359	0.029000	8.620038
N	1.155327	0.028652	7.887215
B	-0.125217	0.036750	-0.153331
N	1.135080	0.036443	-0.842379
B	2.415417	0.034884	-0.153531
N	3.684133	0.034584	-0.849834
B	4.952892	0.033049	-0.153570
N	4.952056	0.031706	1.310675
B	6.217191	0.030133	2.039065
N	6.217230	0.028791	3.500445
B	7.482772	0.027215	4.231172
N	7.480925	0.025925	5.691064
B	8.749378	0.024256	6.422464
N	8.718094	0.022952	7.869367
B	-2.651227	0.034753	4.222183
N	-2.617767	0.033456	5.658147
N	-0.068784	0.027743	10.073301
B	1.158042	0.026154	10.820307
N	6.233219	0.032705	-0.842400
B	7.493528	0.031129	-0.153368
N	7.492270	0.029817	1.307872
B	8.750572	0.028165	2.037853
N	8.753515	0.026869	3.492573
B	10.019602	0.025232	4.222106
N	9.986109	0.023986	5.658093
H	6.252052	0.033617	-1.861965
H	8.528660	0.030938	-0.770272
H	9.793468	0.027955	1.435698
H	11.071390	0.024915	3.634043
H	10.878469	0.022806	6.151591
H	-3.703025	0.036072	3.634112
H	8.310761	0.020834	10.599355
H	6.227054	0.021241	12.025186
H	-1.160403	0.038041	-0.770158
H	-2.425101	0.037009	1.435779
H	3.684225	0.023182	12.017770
H	1.141364	0.025158	12.025233
H	-0.942382	0.027915	10.599326
H	-2.232733	0.030786	8.379282
H	-3.510132	0.033682	6.151649
H	1.116241	0.037346	-1.861950
H	3.684181	0.035509	-1.869508
H	9.601174	0.021816	8.379138
Co	3.566591	1.446913	6.514290
Co	3.359146	3.937873	6.559967

Structure 4:

N	-0.123944	0.035434	1.307903
B	1.151101	0.033835	2.039127
N	2.416284	0.033562	1.310729
B	3.684173	0.031954	2.039139
N	3.684179	0.030689	3.501238
B	4.950284	0.029106	4.231979
N	4.950012	0.027782	5.693862
B	6.216152	0.026140	6.424941
N	6.213078	0.024822	7.887164
B	7.480777	0.023250	8.619997
N	7.437200	0.021965	10.073273
B	6.210385	0.022256	10.820261
N	4.945504	0.023901	10.088644
B	3.684210	0.024207	10.813500
N	2.422912	0.025838	10.088663
B	2.418621	0.027117	8.618847
N	3.684202	0.026757	7.888162
B	4.949774	0.025191	8.618809
B	-1.382221	0.035696	2.037926
N	-1.385158	0.034455	3.492657
B	-0.114401	0.032850	4.231253
N	1.151104	0.032510	3.500463
B	2.418087	0.030974	4.231998
N	2.418356	0.029697	5.693890
B	1.152237	0.029913	6.424956
N	-0.112534	0.031496	5.691131
B	-1.381028	0.031822	6.422547
N	-1.349694	0.030534	7.869427
B	-0.112359	0.029000	8.620038
N	1.155327	0.028652	7.887215
B	-0.125217	0.036750	-0.153331
N	1.135080	0.036443	-0.842379
B	2.415417	0.034884	-0.153531
N	3.684133	0.034584	-0.849834
B	4.952892	0.033049	-0.153570
N	4.952056	0.031706	1.310675
B	6.217191	0.030133	2.039065
N	6.217230	0.028791	3.500445
B	7.482772	0.027215	4.231172
N	7.480925	0.025925	5.691064
B	8.749378	0.024256	6.422464
N	8.718094	0.022952	7.869367
B	-2.651227	0.034753	4.222183

N	-2.617767	0.033456	5.658147
N	-0.068784	0.027743	10.073301
B	1.158042	0.026154	10.820307
N	6.233219	0.032705	-0.842400
B	7.493528	0.031129	-0.153368
N	7.492270	0.029817	1.307872
B	8.750572	0.028165	2.037853
N	8.753515	0.026869	3.492573
B	10.019602	0.025232	4.222106
N	9.986109	0.023986	5.658093
H	6.252052	0.033617	-1.861965
H	8.528660	0.030938	-0.770272
H	9.793468	0.027955	1.435698
H	11.071390	0.024915	3.634043
H	10.878469	0.022806	6.151591
H	-3.703025	0.036072	3.634112
H	8.310761	0.020834	10.599355
H	6.227054	0.021241	12.025186
H	-1.160403	0.038041	-0.770158
H	-2.425101	0.037009	1.435779
H	3.684225	0.023182	12.017770
H	1.141364	0.025158	12.025233
H	-0.942382	0.027915	10.599326
H	-2.232733	0.030786	8.379282
H	-3.510132	0.033682	6.151649
H	1.116241	0.037346	-1.861950
H	3.684181	0.035509	-1.869508
H	9.601174	0.021816	8.379138
Co	3.640194	1.426064	4.980450
Co	2.378505	1.425073	7.143008
